# Production of a Recombinant Fibrinolytic Protease from an Isolate of Serratia marcescens from the Amazon Basin

**DOI:** 10.1021/acsomega.5c02194

**Published:** 2025-05-14

**Authors:** Thayana Cruz Souza, Marcos Gustavo Araujo Schwarz, Paloma Rezende Corrêa, Marília Alves Figueira Melo, Adolfo Jose Mota, Ormezinda Celeste Cristo Fernandes, Leila Mendonça-Lima, Wim Degrave

**Affiliations:** † Instituto Leônidas e Maria Deane – ILMD, 37903 Fiocruz-Amazonas, Rua Teresina, 476, Adrianópolis, Manaus AM 69057-070, Brazil; ‡ Laboratório de Biologia Molecular Aplicada à Micobactérias, Instituto Oswaldo Cruz, Fiocruz, Rio de Janeiro 21040-900, Brazil; § Laboratório de Genômica Aplicada e Bioinovações, Instituto Oswaldo Cruz, Fiocruz, Rio de Janeiro 21040-900, Brazil; ∥ Laboratório de Biodegradação, 67892 Universidade Federal do Amazonas, UFAM. Av. Rodrigo Octávio, 6200, Coroado I, Manaus AM 69080-900, Brazil

## Abstract

Intravenous fibrinolytic agents are essential for the treatment of cardiovascular diseases, acting through plasminogen activation to dissolve thrombi. However, current therapies are often limited by low fibrin specificity, high production costs, and side effects such as bleeding and allergic reactions. In this study, we describe a novel fibrinolytic protease, rSM519, derived from Serratia marcescens CBAM 519 and recombinantly expressed in Escherichia coli. The enzyme was purified via affinity chromatography and exhibited a molecular mass of 56 kDa. Biochemical assays revealed that rSM519 is a metalloprotease with optimal activity at pH 9.0 and 37 °C, significantly enhanced by Mn^2+^ ions. Unlike conventional agents such as tissue plasminogen activator or streptokinase, rSM519 acts directly on fibrin without requiring plasminogen activation. It efficiently degraded the α and β chains of fibrinogen, mimicking plasmin-like behavior, and showed no hemolytic activity. These features position rSM519 as a promising thrombolytic candidate with potential advantages over existing therapies, including lower production costs, reduced side effects, and direct fibrin-targeting activity.

## Graphical abstract

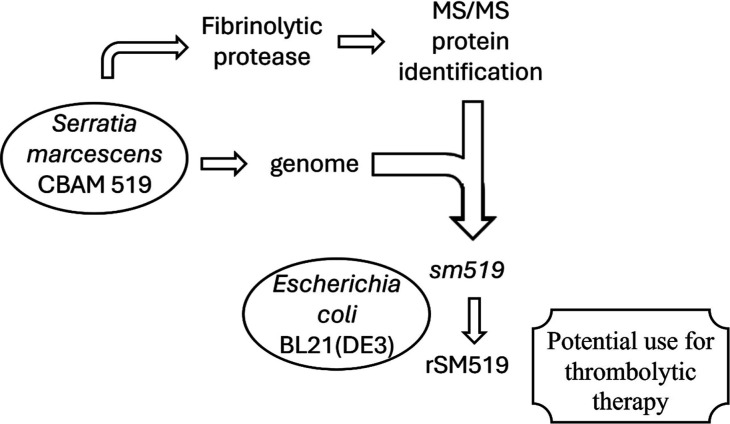

## 1 Introduction

Cardiovascular diseases remain the leading cause of mortality worldwide, and fibrinolytic therapy plays a critical role in managing thrombotic events by promoting clot dissolution through plasminogen activation.^1^ Existing fibrinolytic agents fall into three main categories: (i) plasminogen activators, such as tissue plasminogen activator (tPA), urokinase (uPA), and streptokinase (SK); (ii) plasmin-like enzymes that directly degrade fibrin; and (iii) oral anticoagulants that act via thrombin or factor Xa inhibition.^2–6^

Despite their clinical utility, current fibrinolytic therapies present significant limitations, including low specificity for fibrin, the risk of bleeding, allergic reactions, and high production costs. Moreover, the requirement for plasminogen activation in most therapies may contribute to undesired effects, such as platelet activation and systemic fibrinolysis.^3,4^ In this context, direct-acting fibrinolytic enzymes—particularly those that can be recombinantly produced at low cost—are highly desirable alternatives.^2–4^

Thus, the introduction of new recombinant thrombolytic drugs derived from microorganisms may help overcome some of the shortcomings of the currently available agents. The biochemical efficacy and commercial viability of tPAs produced using recombinant DNA technologies have been demonstrated in several studies employing different expression systems.^3,5,7^

Microbial proteases, especially those from Bacillus species, have been extensively explored for fibrinolytic applications.^8,9^ Nattokinase, due to its high activity and therapeutic applicability in various diseases, has been extensively studied in this context. However, the limited market availability of nattokinase capsules is attributed to low yield and the complexity of enzyme purification from Bacillus subtilis natto.^10,11^

Reports on recombinant fibrinolytic proteases from Serratia species, however, remain scarce, and the few available examples often show low expression yields or result in inactive recombinant products.^12–14^ The efficient expression of active fibrinolytic enzymes in heterologous hosts—especially in Escherichia coli—remains a major technical challenge.^7,15,16^

In this study, we report the cloning, heterologous expression, and functional characterization of a novel fibrinolytic enzyme, rSM519, from Serratia marcescens CBAM 519, an isolate from the Amazon region. This protease exhibits plasmin-like fibrinolytic activity without the need for plasminogen activation and displays a fibrinogenolytic profile characterized by rapid degradation of the α and β chains of fibrinogen while sparing the γ chain. Moreover, rSM519 activity is strongly enhanced by the presence of Mn^2+^, a feature rarely described in comparable enzymes.

Sequence analysis reveals that while SM519 shares partial identity with other Serratia proteases, such as P07268 ^12^ and D4E064,^17^ it diverges significantly in calcium-binding motifs and fibrin degradation patterns—highlighting its novelty among known serralysin-like proteases. These combined features support the potential of rSM519 as a candidate for thrombolytic therapy and for industrial applications requiring specific fibrin degradation.

## 2 Methodology

### 2.1 Materials

Bovine fibrinogen, bovine thrombin, human plasminogen, azocasein, phenyl-methylsulfonyl fluoride (PMSF), ethylenediamine tetraacetic acid (EDTA), pepstatin A, and trichloroacetic acid (TCA) were purchased from Sigma-Aldrich. HisTrap immobilized metal affinity chromatography (IMAC) HP column was obtained from GE Healthcare. PrimeSTAR GXL DNA polymerase was purchased from Takara Bio, and Anza NcoI, Anza XhoI, and T4 DNA ligase were purchased from ThermoFisher Scientific.

### 2.2 Microorganism—Cultivation and Secreted Protein Fraction Production

S. marcescens CBAM 519 was obtained from the Amazon Bacterial Collection (CBAM/FIOCRUZ-Amazonas, Brazil) and cultivated as previously described.^18^ Briefly, the strain was initially cultured on nutrient agar plates at 37 °C for 24 h. Subsequently, an inoculum adjusted to an optical density of approximately 0.2 at 600 nm (OD600) was transferred to 250 mL Erlenmeyer flasks containing 100 mL of medium. The composition of the medium was as follows (w/v): 0.2% KH_2_PO_4_, 0.1% (NH_4_)_2_SO_4_, 0.1% MgSO_4_·7H_2_O, 0.09% NaH_2_PO_4_·H_2_O, 0.1% yeast extract, and 0.5% gelatin. Cultivation was carried out at 37 °C for 24 h under agitation at 150 rpm using a rotary shaker. Following incubation, the cultures were centrifuged at 8000*g* for 10 min at 4 °C, and the resulting supernatant was filtered through a 0.22 μm membrane.

### 2.3 Mass Spectrometry for Protein Identification

Total supernatant proteins from 1.5 mL of a 24 h culture of S. marcescens on gelatin-containing medium were precipitated with TCA (final concentration ∼17% [w/v]) and resuspended in 50 μL of IEF buffer (7 M urea, 2 M thiourea, and 4% (w/v) CHAPS). Total protein concentration was measured with a NanoDrop equipment, and different amounts of sample (5, 10, 5, and 20 μg of total protein) were analyzed by SDS-PAGE 12%, followed by CBB-R250 staining. Bands were excised from the gel, and proteins were trypsinized, as described.^19^ Tryptic peptides were desalted and concentrated with C18 ZipTip, following the manufacturer’s protocol.

Samples were analyzed by MALDI-TOF/TOF mass spectrometry on an AB SCIEX TOF/TOF 5800 equipment. Mass spectra were acquired in positive ion reflectron mode, calibrated with an external peptide mixture mass standard. Upon MS/MS analysis, fragment peptides were generated by postsource decay, with the collision gas chamber off, from the ten most abundant peptides of each MS analysis.

Both MS and MS/MS spectra were used for protein identification using Mascot software. Search was performed against the NCBI-nr nonredundant database, without taxon restriction, and parameters were trypsin as the enzyme of choice and one missed cleavage, ±1 Da for the precursor mass, and ±0.5 Da for the fragment ion mass. Oxidation of methionines along with N-terminal acetylation of proteins, N-terminal formylation, deamidation, and cyclization of glutamine (pyro-glutamate) were allowed as possible modifications, whereas alkylation of cysteines (carbamidomethylcysteines) was set as constant modification.

### 2.4 Genome Sequencing, Assembly, and Annotation

The bacterial DNA was extracted using a Blood and Cell Culture DNA Mini kit (Qiagen), preceded by cell lysis with zirconium beads in a bead beater apparatus (Biospec Products INC.), according to the manufacturer’s recommendations.

DNA purity was assessed using the NanoDrop 2000 spectrophotometer (Thermo Scientific), evaluating the 260/280 and 260/230 nm absorbance ratios. Subsequently, DNA quantification was carried out using the Qubit 2.0 fluorometer with the Qubit ds DNA BR kit (Invitrogen, Life Technologies). This qualified and quantified the genomic DNA to be used for library preparation using an IonXpress Plus gDNA Fragment Library kit (ThermoFisher). Sequencing was conducted using the Ion PGM Hi-Q View Sequencing kit on 850 run flows. The run pertaining to the S. marcescens CBAM 519 sample yielded 522,203 reads with an average size of 257 bases, and data interpretation achieved an utilization rate of 63% (TorrentSuite 5.4.0 software).

Genome assembly of S. marcescens CBAM 519 was conducted using AssemblerSpades v. 5.6.01 following a de novo strategy (reference-free assembly), therefore minimizing bias toward conserved regions, enabling the detection of strain-specific features, as well as ensuring comprehensive reconstruction of potentially novel genetic elements. Genome annotation was performed using the RAST server (http://rast.nmpdr.org/) and PATRIC (https://www.patricbrc.org/) to identify and annotate sequences with biological functions.

### 2.5 Cloning, Expression, and Purification of the Gene Encoding the Protease from S. marcescens

Peptides presumably derived from the S. marcescens CBAM 519 protease were used to search for proteins deposited in the NCBI database. The protein sequence showing the highest similarity (GenBank accession number BAO08779.1) was then compared using NCBI’s tblastn tool with the contigs obtained from the assembled genome sequence.

The gene *sm519* encoding the protein from S. marcescens CBAM 519 was cloned into the pET28a­(+) vector, including code for a C-terminal 6-His tag. Genomic DNA from S. marcescens was used as the template for amplification, using the Smarc-pETFOR primer (TCTCCATGGAATCTACTAAAAAGGCAATT) and Smarc-pETREV primer (ACGTCTCGAGCACGATAAAGTCGGTGGC) and PrimeSTAR GXL DNA polymerase (Takara Bio), following the manufacturer’s protocol.

The plasmid and insert were digested with Anza NcoI and Anza XhoI enzymes (ThermoFisher Scientific), followed by ligation using T4 DNA ligase (Invitrogen) according to the manufacturer’s instructions. The 5-fold diluted reaction was used to transform electrocompetent E. coli TOP10 cells using a GenePulser apparatus (25 μF, 2.5 kV, 200 Ω), followed by incubation on LB agar supplemented with kanamycin (25 μg/mL).

After confirming the expected construction by colony PCR and Sanger sequencing, plasmid DNA was extracted with a QIAprep spin miniprep kit (Qiagen) and was used to transform E. coli BL21 (DE3) cells. Expression was induced by adding 1 mM IPTG and incubating at 37 °C for 3 h.

The bacterial pellet was resuspended in lysis buffer (20 mM Tris–HCl pH 7.5, 300 mM NaCl, 0.5% v/v Triton X-100) and lysed using a bead beater with 3 pulses of 1 min each, with 1 min intervals on ice. The total lysate was recovered and centrifuged (15,000*g* for 10 min), and the insoluble inclusion bodies were washed three times with wash solution (50 mM Tris–HCl pH 8.5, 0.5% v/v Triton X-100, 5 mM EDTA, 150 mM NaCl). The insoluble proteins were then resuspended in 8 M urea, 0.3 M NaCl, and 50 mM Tris–HCl pH 8.5. Prior to purification, the protein solution was diluted to 4 M urea and clarified by centrifugation.

Purification was carried out using IMAC with a 1 mL HisTrap IMAC HP column (GE Healthcare) on an KTA Purifier chromatography system at room temperature, with the absorbance monitored at 280 nm. Elution was performed in steps of 5 column volumes: 10%, 20%, 30%, 40%, and 100% of elution buffer (same as binding buffer [100 mM Tris–HCl pH 7.5, 300 mM NaCl, and 5 mM imidazole] but with 0.5 M imidazole). Fractions containing the recombinant protein were pooled, and the buffer was exchanged with PBS 1× using an Amicon system.

### 2.6 S. marcescens Fibrinolytic Enzyme Sequence In Silico Analysis

The in silico translation and analysis of the deduced amino acid sequences were performed using the ExPASy Proteomics (Expert Protein Analysis System: http://us.expasy.org/tools) Translate tool from the Swiss Institute of Bioinformatics (SIB). The virtual SignalP program (http://www.cbs.dtu.dk/services/SignalP) was used to estimate the cleavage sites between the signal peptides (pro-peptide) and the mature fibrinolytic enzyme. Multiple alignments of the amino acid sequences were conducted using the UniProtKB/Swiss-Prot platform (https://www.expasy.org/resources/uniprotkb-swiss-prot). The putative catalytic residues were predicted by ScanProsite (https://prosite.expasy.org/). The physicochemical properties of SM519, such as amino acid composition, isoelectric point (pI), and theoretical molecular weight, were calculated using the ExPASy ProtParam Tool (http://us.expasy.org/tools/protparam.html).

### 2.7 Fibrinolytic Assays

#### 2.7.1 Determination of Fibrinolytic Activity

The fibrinolytic activity was determined according to Wang et al.^20^ First, 0.4 mL of 0.72% fibrinogen was added to a test tube with 0.1 mL of 245 mM phosphate buffer (pH 7) and incubated at 37 °C for 5 min. Then, 0.1 mL of a 20 U/mL thrombin solution was added and incubated at 37 °C for 10 min followed by the addition of 0.1 mL of diluted enzyme solution, and incubation was continued at 37 °C. This solution was homogenized again after 20 and 40 min. At 60 min, 0.7 mL of 0.2 M TCA was added and mixed. The reaction mixture was centrifuged at 15,000*g* for 10 min. Then, 1 mL of the supernatant was collected, and the absorbance at 275 nm was measured. In this assay, one unit (fibrin degradation unit per L) of enzyme activity is defined as an increase in absorbance of 0.01 per minute at 275 nm of the reaction solution. All experiments were performed in triplicate.

#### 2.7.2 Fibrin Zymography

Fibrinolytic activity was analyzed on a fibrin zymography gel as previously described.^21^ Briefly, fibrinogen (0.12% w/v) and thrombin (1 U/mL, both from Sigma) were mixed with a 12% polyacrylamide gel solution, and electrophoresis of protease solution was carried out. The gel was then washed with 2.5% Triton X-100 for 1 h, rinsed twice with distilled water, and incubated in the reaction buffer (0.1 M glycine, pH 8.4) at 37 °C for 18 h. The gel was stained with Coomassie Blue R-250 for 1 h and then destained. The digested bands were visualized as the nonstained regions of the fibrin gel. The molecular mass was calibrated using a Broad Range protein marker (BioRad).

#### 2.7.3 Determination of Plasminogen Activation Capacity

Fibrinolytic activity was assessed using both plasminogen-free and plasminogen-rich fibrin plate methods, with minor modifications.^22^ For the preparation of plasminogen-free fibrin plates, a fibrinogen solution (5 mL of 0.5% human fibrinogen in 20 mM Tris–HCl buffer, pH 7.4) was mixed with 20 U of thrombin solution and 5 mL of 1% agarose in Petri dishes. These plates were then heated at 80 °C for 30 min. In contrast, plasminogen-rich fibrin plates, containing 5 U of plasminogen, were not subjected to heating. To conduct the assay, 10 μL of the sample solution was carefully applied to wells (Ø = 5 mm) created in the fibrin plates and incubated at 37 °C for 18 h. Fibrinolytic activity was evidenced by the formation of a clear, transparent zone, where fibrin was hydrolyzed. The diameter of this zone is directly proportional to the fibrinolytic activity. The difference in the area of lytic zones between the two types of plates indicates the presence of plasminogen activator activity. All experiments were performed in triplicate to obtain average measurements of the zone diameters.

#### 2.7.4 Fibrinogenolytic Assay

Fibrinogenolytic activity was determined as follows: 150 μL of 1.0% human fibrinogen solution was incubated with 50 μL of the purified enzyme at 37 °C for different times (1, 5, 10, 15, 30, 60, and 120 min).^23^ The reaction was stopped by the addition of a denaturing buffer. The digested products were analyzed by 12% SDS-PAGE according to the method of Laemmli.^24^

The fibrinolytic activity corresponding to the alpha, beta, and gamma chains was quantified using the mean gray value obtained through ImageJ software (version 2.0.0; National Institutes of Health, Bethesda, MD, USA). Briefly, to analyze the decay of fibrinolytic activity for each individual chain, the control time point was designated as representing 100% fibrinolytic activity with subsequent time points expressed as a percentage relative to this initial value. For the analysis aimed at assessing the contribution of each chain to the total fibrinolytic activity, the sum of the mean gray values of the alpha, beta, and gamma chains at the control time point was considered to represent 100% total activity. All subsequent measurements were expressed relative to the baseline total activity.

### 2.8 In Vitro Hemolysis Assay

Hemolysis assay was performed according to the method previously described with some modifications.^25^ The blood agar plate made of blood agar base and fresh defibrinated sheep blood was prepared for the hemolysis assay. The purified enzyme (20 μL of a 16 μg/μL solution) was placed into the previously punched holes in the blood agar plate (Ø = 5 mm). After incubation at 37 °C for 3 days, the plate was examined for the presence of translucent halos. This experiment was performed in triplicate.

### 2.9 Characterization of the Recombinant Fibrinolytic Protease

#### 2.9.1 Effect of pH on Protease Activity and Stability

The effect of pH on protease activity was estimated by enzymatic assays with buffers ranging from pH 5.0 to 9.0. To assess the influence of pH on proteolytic activity, both the reaction and blank systems were prepared using azocasein as the substrate in the following buffer solutions: 0.1 M citrate buffer (pH 4–6), 0.1 M Tris–HCl buffer (pH 7–8), and 0.1 M sodium carbonate/bicarbonate buffer (pH 9).^21^ Reactions were incubated for 1 h at 25 °C, and proteolytic activity was determined by the method previously described.^18^ For evaluation of the enzyme stability, the enzymatic extract was dispersed (1:1) in the buffers (pH 5.0 to 9.0) and maintained at 25 °C for 24 h. Residual proteolytic activity was determined under the standard assay conditions. All experiments were performed in triplicate.

#### 2.9.2 Effect of Temperature on Protease Activity and Stability

The optimal temperature was determined by incubating the enzyme at different temperatures ranging from 25 to 80 °C, followed by the activity assay at the pH determined as optimal. For thermal stability, the enzyme was incubated at different temperatures ranging from 25 to 80 °C for 1 h, and the proteolytic activity was determined according to the optimal conditions of pH and temperature. All samples were prepared in triplicate.

#### 2.9.3 Effect of Inhibitors on Protease Activity

The effect of inhibitors on enzyme activity was evaluated in the presence of the following compounds: 10 mM methylphenylsulfonyl fluoride (PMSF), 10 mM 2-mercaptoethanol, 10 mM ethylenediaminetetraacetic acid (EDTA), and 1 mM pepstatin A. All assays were done in triplicate.

#### 2.9.4 Effect of Metal Ions on Protease Activity

The protease activity was evaluated in the presence of the following metal ions, at a concentration of 10 mM: zinc (Zn^2+^), magnesium (Mg^2+^), copper (Cu^2+^), iron (Fe^2+^), calcium (Ca^2+^), manganese (Mn^2+^), sodium (Na^+^), and potassium (K^+^) and incubated at 37 °C for 60 min. The protease was incubated for 60 min at 37 °C in a 50% (v/v) solution containing the respective ion. The residual activity was measured as a percentage of the proteolytic activity in the sample, compared to a control sample without any inhibitors. All samples were prepared in triplicate.

### 2.10 Statistical Analysis

Data were expressed as mean ± standard deviation (SD) and statistically analyzed using the Student’s *t*-test. The number of replicates is indicated in the caption of the respective figures.

## 3 Results and Discussion

### 3.1 Production of the Recombinant Fibrinolytic Enzyme

#### 3.1.1 Protein Identification by Mass Spectrometry

The fibrinolytic protease from S. marcescens CBAM 519 which was previously characterized in its native form in our lab^18^ and visualized as a prominent band of approximately 56 kDa on SDS-PAGE was analyzed by mass spectrometry (Figure 1). From the obtained spectra, a search against proteins deposited in the NCBI database revealed similarity with 4 peptide fragments (Table 1) matching metalloproteases from the serralysin subfamily of S. marcescens (GenBank accession number BAO08779.1). This protein has a predicted molecular mass of 52 kDa, similar to the value found for the fibrinolytic enzyme of S. marcescens CBAM 519.

**Figure 1:**
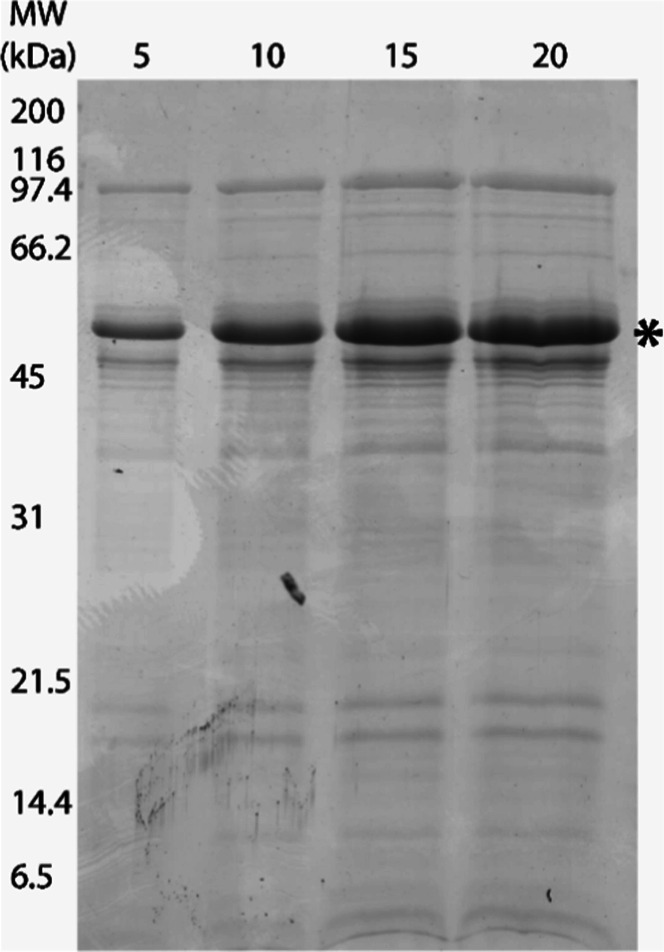
Secreted proteins of Serratia marcescens CBAM 519 after 24 h of growth in medium containing gelatin (protease induction). After TCA precipitation and resuspension in IEF solution, the total protein content was measured using a Nanodrop (280 nm) and applied onto 15% SDS-PAGE (5, 10, 15, and 20 μg, as indicated above the lanes). Coomassie R 250 stain was used for visualization. The most abundant band (∼56 kDa) was excised and analyzed by mass spectrometry.

**Table 1:** Protein Identification in the 56 kDa Band of S. marcescens CBAM 519 Secretome

identified protein/species	protein score	theorical MW (kDa)	coverage		identified peptides by ms/ms		
				Sequence	*m*/*z*	Ion score	*E*-value
serralysin metalloprotease [Serratia marcescens]—NCBI accession code: BAO08779.1	507	52.759	13%	TGYDAVDDLLHYHER	1488.761	134	5.4 × 10^–7^
				DSFSNEQAGLFITR	1584.861	138	2.4 × 10^–7^
				TGDTVYGFNSNTGR	1759.951	102	0.00099
				VIFAAWDAGGNDTFDFSGYTANQR	2665.424	140	3.1 × 10^–7^

#### 3.1.2 Sequence Analysis of the Fibrinolytic Protease from S. marcescens CBAM 519

Search for the corresponding genome sequence using the peptide sequences obtained above led to the tentative identification of the *sm519* gene encoding the fibrinolytic enzyme of S. marcescens CBAM 519 (SM519). This Whole Genome Shotgun project has been deposited at DDBJ/ENA/GenBank under accession JBLZWK000000000. The version described in this paper is version JBLZWK010000000. Analysis of the *sm519* gene and its flanking DNA regions revealed the presence of an open reading frame of 1444 bp, encoding a protein of 487 amino acids.

The predicted physicochemical properties for this protein were obtained using the ExPasy and ProtParam tools. SM519 has a predicted molecular weight of 52.28 kDa and an isoelectric point (pI) of 4.67, close to the apparent molecular weight of the purified enzyme (56 kDa), determined by SDS-PAGE. The total number of negatively charged residues (Asp + Glu: 54) exceeded the total number of positively charged residues (Arg + Lys: 29). With a GRAVY index of −0.425, the protein exhibits hydrophilic characteristics. The instability index was calculated as 26.55, indicating that the enzyme is stable. SM519 showed a high aliphatic index of 66.59, suggesting a reasonable predicted thermal stability for the protein.

The deduced amino acid sequence encoded by the *sm519* gene was compared with other known proteases from Serratia species (Figure 2), revealing high levels of identity: 97% identity with the sequence of protease P07268 from S. marcescens ATCC 21074/E-15; 91% with serralysin A0A240AD01 from Serratia ficaria; and 80% with D4E064 from Serratia odorifera. Interestingly, the amino acid sequence of SM519 showed low identity with other reported fibrinolytic proteases, such as nattokinase (14.2%), streptokinase (10.94%), urokinase (8.87%), and plasmin (8.21%). These findings suggest that the SM519 enzyme from S. marcescens CBAM 519 is a novel fibrinolytic enzyme family. Furthermore, differences were observed in the sequence of the third calcium-binding box, while the catalytic and zinc-binding sites were identical across the analyzed sequences. Alignment with sequences of previously characterized proteases enables the prediction of potential similarities between our target enzyme and known enzymes, thereby guiding preliminary characterization experiments. Moreover, the identification of sequence differences helps pinpoint regions of interest that may confer novel functional properties. Such unique features could prove biologically or therapeutically relevant, potentially offering solutions to unmet medical needs, such as improved fibrinolytic therapies.

**Figure 2:**
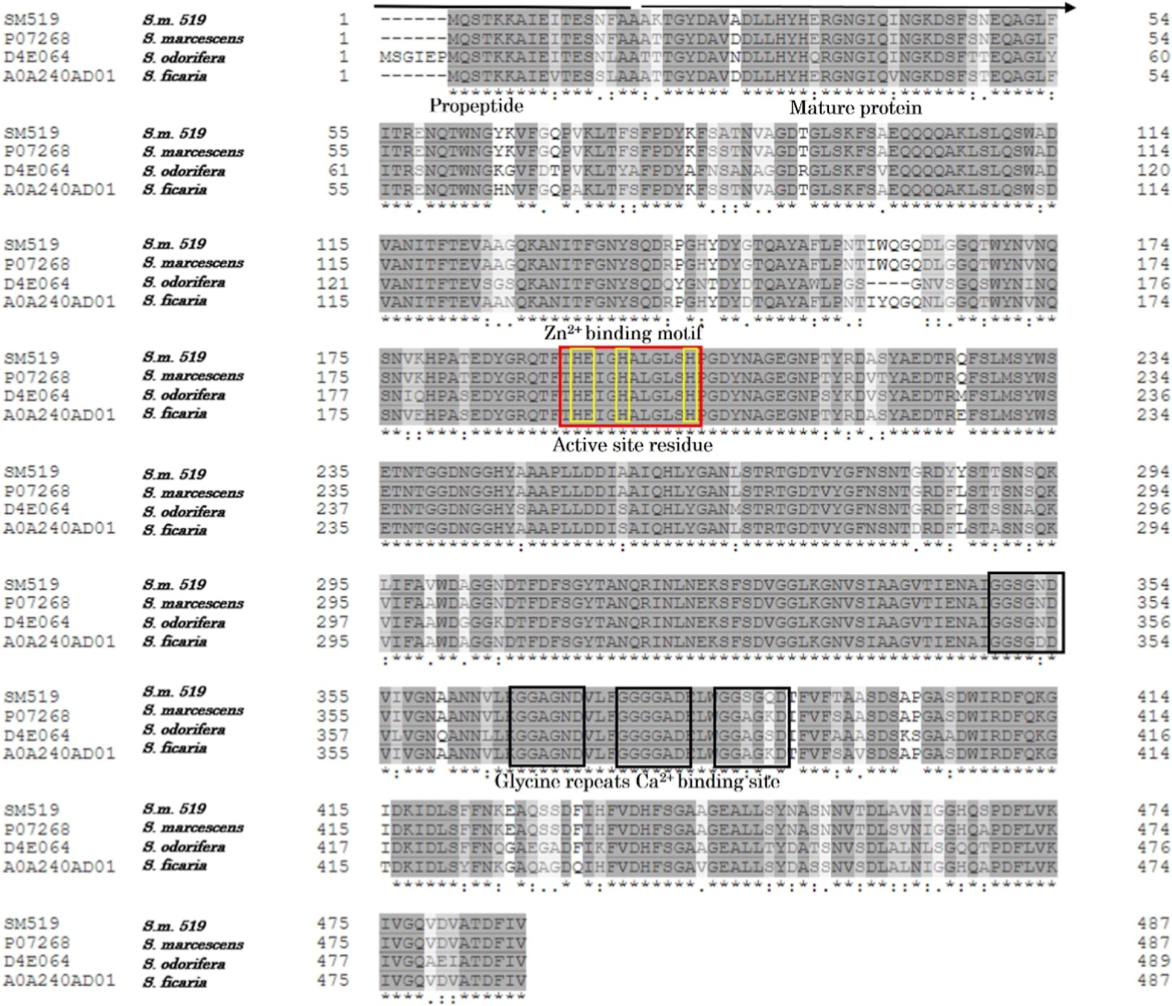
Comparison of SM519 with other Serratia proteases. The amino acid sequence of the fibrinolytic protease from S. marcescens CBAM 519 was compared with proteases P07268 from S. marcescens ATCC 21074/E-15 (accession number: 617), D4E064 from S. odorifera (accession number: 2647522), and A0A240AD01 from S. ficaria (accession number: 61651). Identical residues are shaded in dark gray (*), conserved residues are shaded in medium gray (:), and light gray indicates semiconservative substitutions (.). Yellow-highlighted characters denote amino acid residues of the enzyme’s active site. Zinc and calcium-binding domains (glycine-rich repeat) are indicated. Numbers written on both sides of the lines indicate the positions of amino acids. Putative residues of the pro-peptide and mature protease are also indicated.

ScanProsite and InterPro analyses revealed several conserved motifs and domains in the SM519 enzyme. Specifically, SM519 contains a conserved Zn^2+^-binding motif (HEXXHXUGUXH: where X denotes any amino acid, and U denotes a hydrophobic amino acid), a calcium-binding domain characterized by the presence of four glycine-rich repeats (GGXGXD), and lack of cysteine residues. These characteristics are typical of metalloproteases belonging to the serralysin family (peptidase M10, subfamily M10B; Figure 2). Serralysins and related proteases are important virulence factors in pathogenic bacteria.^26^ Most microbial fibrinolytic proteases identified to date have been classified as serine proteases resembling subtilisin, belonging to the peptidase S8 family.^27^

The analysis by SignalP indicated the presence of an N-terminal signal peptide (pro-peptide) consisting of 16 amino acid residues in the SM519 fibrinolytic enzyme. The exact role of the pro-peptide region is not fully understood but is thought to assist in the secretion process and proper folding of the mature enzyme.^26^ The “pro” sequence is subsequently autocatalytically cleaved to form the active enzyme. The pro-peptide is presumed to be removed in later stages of the secretion process.^27^

#### 3.1.3 Heterologous Expression of the Recombinant Enzyme

To express SM519, the corresponding gene was cloned into the pET28a­(+) vector and subsequently introduced into the E. coli BL21 (DE3) strain, leading to the efficient expression of the SM519 protease using the pET28a-*sm519* construct. Upon analysis by SDS-PAGE, it was observed that the recombinant fibrinolytic enzyme (rSM519) was expressed in E. coli as insoluble protein aggregates (Figure 3A), specifically as inclusion bodies. However, the enzyme was active after denaturation and renaturation, as confirmed by zymography (Figure 3B). No proteolytic activity was detected in the soluble fraction after bacterial lysis. Therefore, it was necessary to extract the recombinant protein from the inclusion bodies using a denaturing solution containing 8 M urea for solubilization, followed by purification via chromatography.

**Figure 3:**
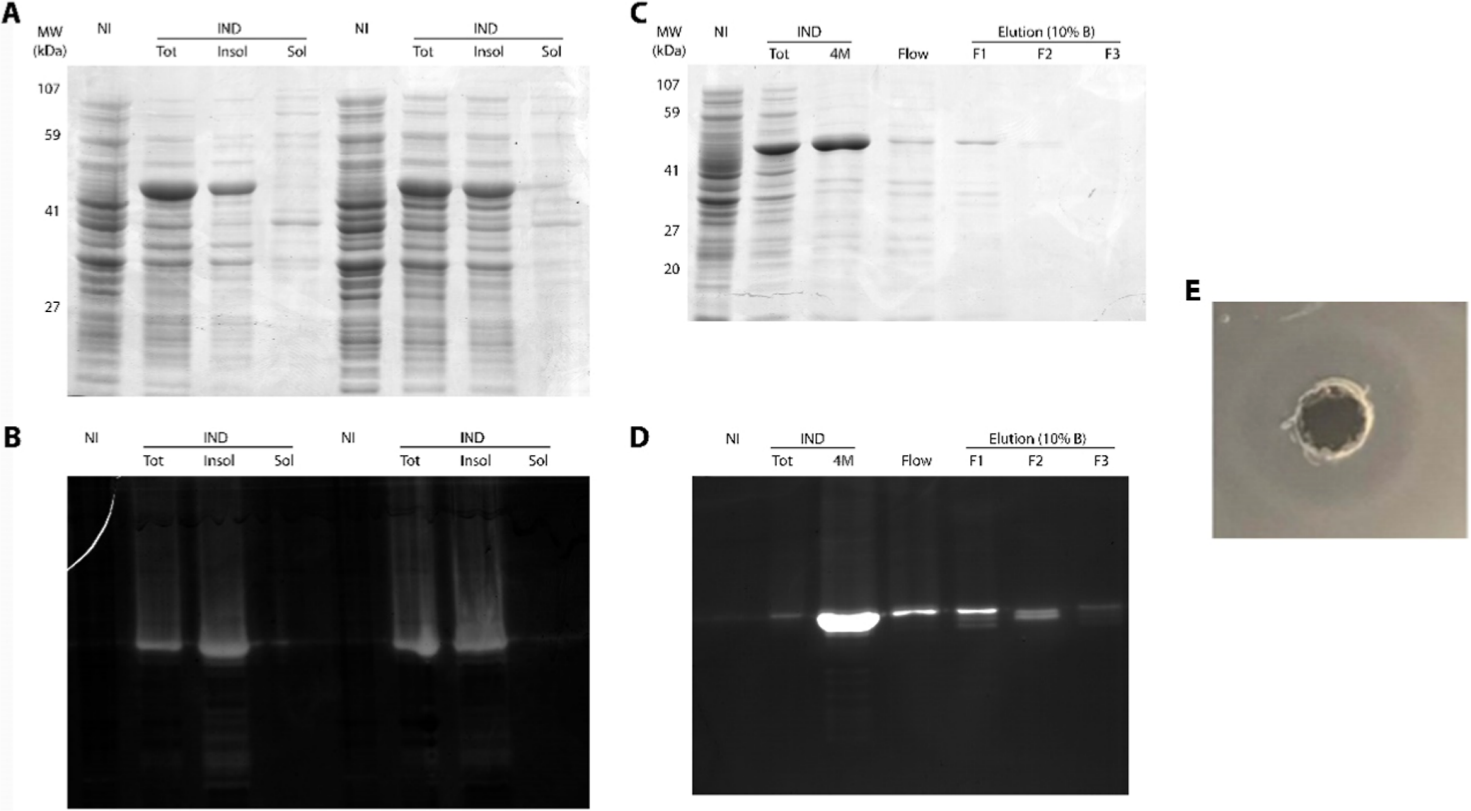
Cloning, expression, and purification of rSM519. (A) Protein extract from induced (IND) or noninduced (NI) cultures harboring pET28a-*sm519* resolved by 12% SDS-PAGE, analyzing total protein (Tot), insoluble (in), and soluble (sol) fractions after lysis and clarification. (C) Proteins resolved by 12% SDS-PAGE, displaying the sample applied to the Ni^2+^-IMAC column (solubilized inclusion-body fraction in 4 M urea), nonbound proteins (flow), and elution fractions with buffer B at 10%. (B) Fibrin zymography before and (D) after purification. (E) Fibrin plate assay of the purified sample obtained from fraction 1.

The purification of rSM519 was achieved in a single chromatographic step and yielded 0.5 mg/mL. A band with an approximate molecular weight of 56 kDa was observed in fraction F1 by SDS-PAGE, which is consistent with the expected size deduced from the amino acid sequence (Figure 3C). The molecular weight of fibrinolytic enzymes of different origins broadly varies from 14 kDa to 97 kDa.^28^ The large-scale preparation of rSM519 as a biocatalyst for biotechnological applications can therefore be pursued.

The fibrinolytic activity of rSM519 was quantified and analyzed by using fibrin zymography and fibrin plate assays. Enzymatic activity was observed exclusively in the insoluble fraction. In Figure 3E, a clear hydrolysis zone is visible using fraction F1, in which a band was observed on zymography. Thus, it was confirmed that the rSM519 protease was successfully expressed in its active form in the insoluble fraction, allowing for recovery without compromising the enzymatic activity (Figure 3E).

Many genes encoding microbial fibrinolytic polypeptides have been cloned and expressed as inactive inclusion bodies in E. coli. Successful refolding of fibrinolytic enzymes from inclusion bodies has been described in only a few studies.^29–31^ For example, nattokinase was produced in a large quantity of insoluble protein in inclusion bodies without enzymatic activity. These authors employed refolding solutions at different pH values to renature the protein, successfully recovering full enzyme activity levels.^30^

The presence of the “pro” sequence facilitates the expression of fibrinolytic enzymes in soluble form in E. coli.^27^ During protein secretion, the signal peptide and pro-peptide are typically removed by host proteases.^32^ When the “pro” sequence was included in the Bacillus amyloliquefaciens gene encoding subtilisin DFE (Trx-Pro-subtilisin DFE), it was partly expressed in soluble form in E. coli and mostly as inclusion bodies; conversely, in the absence of the “pro” sequence (Trx-subtilisin DFE), the enzyme was only present in inclusion bodies and inactive.^31^ This highlights the importance of the pro-peptide in the expression of fibrinolytic enzymes.^33^

B. subtilis ZA400 was isolated from kimchi to obtain the fibrinolytic enzyme sequence BsfA, which was cloned into an E. coli expression system using the vector pET26b­(+), which includes a signal peptide (pelB) before the multiple cloning site that promotes secretion of the recombinant protein to the bacterial periplasm.^32,33^ Therefore, the pET26b-ZA400 construct can express the *bsfA* gene upon IPTG induction to produce BsfA, which can be secreted in a soluble form into the periplasm. The authors proposed using purified BsfA for the treatment of cardiovascular diseases and for developing a new type of functional food with strong fibrinolytic activity. In our study, however, despite the fibrinolytic protease (SM519) having the “pro” sequence, the enzyme was not expressed in soluble form in E. coli but retained its activity after solubilization of the inclusion bodies.

The expression of rSM519 could be enhanced in future studies through optimization tests aimed at maximizing expression by varying parameters such as growth temperature, induction conditions, IPTG concentration, and induction time. Such optimization efforts have the potential to significantly reduce the production and purification costs of the fibrinolytic enzyme, making it a viable alternative for industrial applications. However, it is crucial to recognize that the transition from laboratory-scale to industrial production involves multiple challenges. Key issues include mass and heat transfer limitations in large-scale bioreactors as well as metabolic stress on the host organism at high cell densities. These factors can significantly impact protein expression rates and enzyme properties—including folding efficiency, stability, and activity—necessitating careful optimization at each scale-up stage.

### 3.2 rSM519 Characterization

#### 3.2.1 Plasminogen Activation

The fibrinolytic enzyme rSM519 demonstrated direct action on fibrin without activating plasminogen, as evidenced by the lack of difference in halo diameters between plates with and without plasminogen (data not shown). This suggests that rSM519 exhibits characteristics of a plasmin-like protease, directly degrading fibrin, similar to what we previously observed with the native enzyme from S. marcescens CBAM 519.^19^ This represents an advantage compared to clinically used plasminogen activators such as urokinase (UK), streptokinase (SK), and tPA, as it may avoid side effects such as platelet activation associated with plasmin formation.^34,35^

#### 3.2.2 Fibrinogenolytic Activity

The pattern of fibrinogen degradation by rSM519 was analyzed by using SDS-PAGE (Figure 4). rSM519 exhibited fibrinogenolytic activity, completely degrading the α chain within 5 min followed by degradation of the β chain after 30 min. In contrast to what we previously observed for the native enzyme from S. marcescens CBAM 519,^19^ only the γ chain resisted degradation. The recombinant enzyme shows similarities to plasmin, as plasmin also cleaves the α and β chains of fibrinogen but not the γ chain.

**Figure 4:**
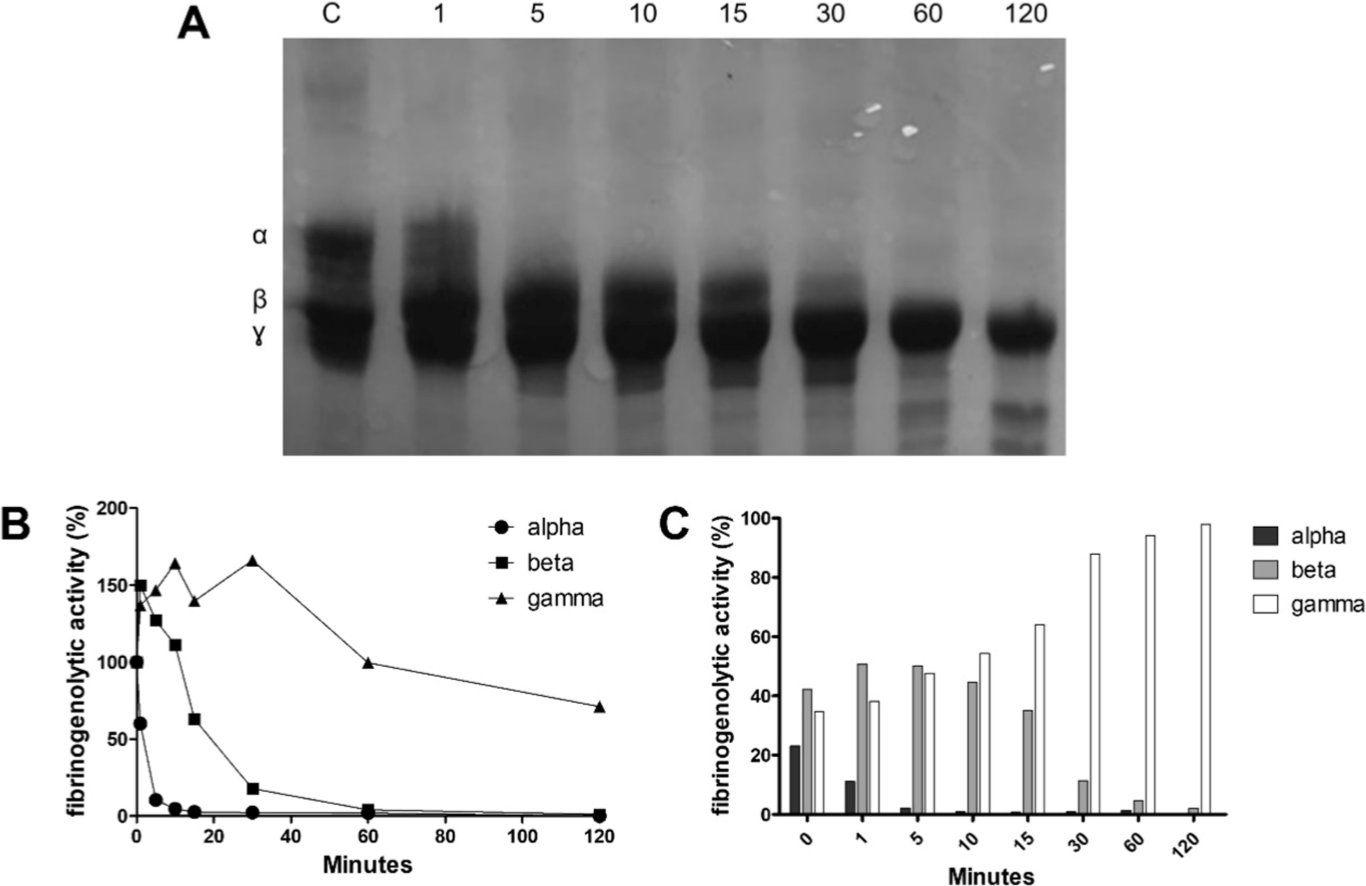
(A) The fibrinogenolytic activity of rSM519 was analyzed in 12% SDS-PAGE stained with Coomassie Brilliant Blue R-250 after specified time intervals of enzyme and fibrinogen coincubation. Line C: control (fibrinogen), 1 to 120: incubation time (min). Bands corresponding to the α, β, and γ chains are shown. (B) The fibrinogenolytic activity was calculated for each chain over time using densitometry and analysis with the ImageJ software. (C) Additionally, the percentage of each chain relative to the total was also determined throughout the kinetic assay.

This degradation profile closely resembles that of nattokinase, a well-characterized fibrinolytic enzyme from Bacillus subtilis, which also preferentially hydrolyzes the α-chain of fibrinogen, followed by partial degradation of the β-chain, with minimal or no effect on the γ-chain.^4,10^ This specific cleavage pattern is therapeutically advantageous as the α-chain plays a central role in fibrin clot stabilization. Its selective degradation leads to effective clot destabilization while preserving the structural integrity of circulating fibrinogen, potentially reducing hemorrhagic risks.^27^ Therefore, enzymes such as rSM519 that mimic plasmin and nattokinase in their fibrinogenolytic behavior represent valuable candidates for safer thrombolytic therapy.

This selective fibrinogenolytic profile is also observed in other microbial serine metalloproteases, such as those from Bacillus circulans CFR11, as well as recombinant fibrinolytic enzymes expressed in E. coli from B. subtilis HK176 ^36^ and Bacillus pumilus BS15 ^37^ expressed in E. coli. Conversely, fibrinolytic enzymes from Streptomycessp. and Bacillussp. degrade only the β and γ chains but not the α chains.^38,39^

In fibrinogen molecules, plasmin-mediated fibrinogenolysis involves cleaving the terminal third of the alpha chains and the initial amino acids of the beta chain. The quest for a fibrinolytic enzyme with specificity for the alpha chain is crucial, as proteolytic degradation of alpha chains results in decreased clot stability.^40^ Lower fibrinogen levels reduce the incidence of thrombosis. These findings suggest that such a protease could be a candidate for use in thrombolytic therapy and prevention of blood clot formation.

On the other hand, comparison with the native enzyme reveals a notable difference, raising questions about potential structural modifications or post-translational processing during heterologous expression. Since this involves production in E. coli, key distinctions from Serratia must be considered, including differences in chaperone systems, physicochemical properties of the bacterial cytosol, and codon usage patterns. These factors may collectively influence production kinetics and protein folding efficiency. Furthermore, the enzyme might undergo specific post-translational modifications in its native host that are either absent or nonfunctional in E. coli, potentially altering its structural characteristics and folding pathway. This peculiarity warrants further investigation to understand the underlying molecular basis of fibrinogen catalysis.

#### 3.2.3 Biochemical Characterization

To evaluate the effect of temperature, fibrinolytic activity was assessed from 25 to 80 °C, with maximum detected enzyme activity defined as 100% (Figure 5). rSM519 exhibited peak activity at 37 °C and showed notable stability between 25 and 40 °C, maintaining over 90% of its activity after 1 h incubation. However, above 60 °C, more than 50% of the activity was lost, indicating a decreased thermal stability at higher temperatures.

**Figure 5:**
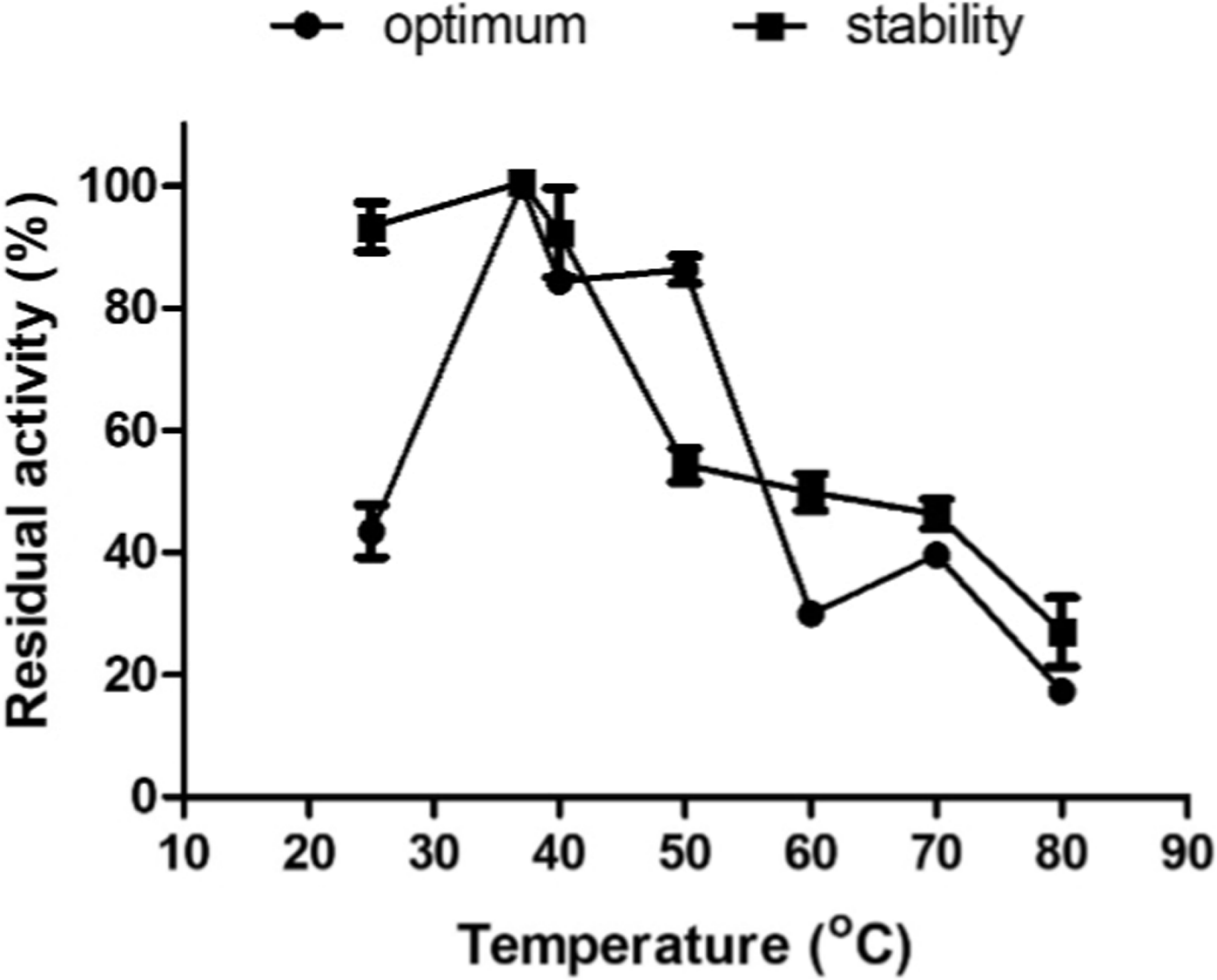
Effect of temperature on the activity of rSM519. () Optimal temperature: the maximum detected activity exhibited by the enzyme was considered 100% relative activity. () Enzyme stability: the effect of temperature on enzyme stability was measured after 1 h of incubation at different temperatures and expressed as a percentage of residual activity. Assays were conducted under optimal enzyme pH conditions (pH 9.0). Values represent the mean ± SD (*n* = 3) from three independent experiments.

Although recombinant fibrinolytic proteases can vary in terms of stability and optimal activity, most exhibit high catalytic efficiency around 37 °C and a significant reduction in activity at higher temperatures, consistent with data obtained for rSM519. The recombinant fibrinolytic protease from Bacillus subtilis also showed maximum activity around 37 °C.^41^ Another study involving a protease from Paenibacillus polymyxa expressed in E. coli demonstrated that the enzyme maintained over 80% of its activity up to 45 °C, but the activity dropped sharply at higher temperatures.^42^

This activity and thermal stability profile are crucial for biotechnological applications, particularly in industrial processes that can be optimized to maximize the enzyme efficiency. Furthermore, these data provide a foundation for future enzymatic modifications aimed at enhancing the thermal stability and efficiency of rSM519.

The enzyme exhibited a maximum activity at pH 9.0, where it was recorded as 100% (Figure 6). Conversely, at pH 4.0, enzymatic activity was severely reduced, highlighting the enzyme’s sensitivity to acidic conditions. In addition to optimal activity, the stability of rSM519 against pH variations was examined. Results showed that the enzyme retained over 70% of its activity over a broad pH range from 5.0 to 9.0. Similar findings were observed with the native enzyme from S. marcescens CBAM 519.^19^ The optimal pH observed for rSM519 aligns with previous studies on microbial fibrinolytic proteases, indicating a preference for neutral to alkaline environments for maximum activity.^28^ It was previously characterized that nattokinase from B. subtilis YF38 expressed in E. coli was stable within pH 6.0–10.0, with optimal fibrinolytic activity at pH 8.0.^34^ These variations can be attributed to structural differences and evolutionary adaptations of enzymes to their microbial environments. Moreover, this robustness across different pH values is an important attribute for biotechnological and therapeutic applications, ensuring that the enzyme can function effectively under varied physiological conditions.

**Figure 6:**
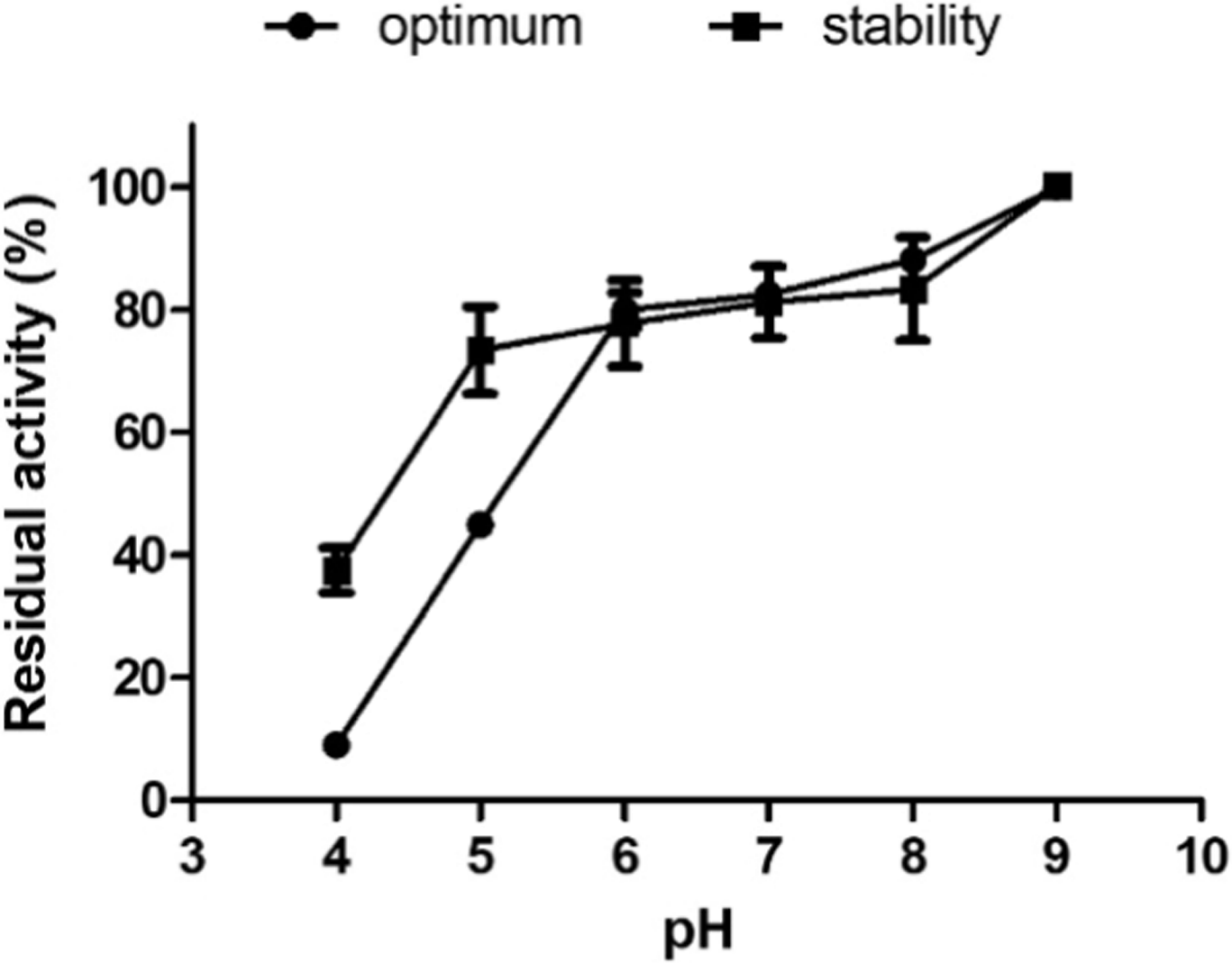
Effect of pH on the activity of rSM519. () Optimal pH: the maximum activity exhibited by the enzyme was considered 100% relative activity. () Enzyme stability: the effect of pH on enzyme stability was measured after 24 h of incubation and expressed as a percentage of residual activity. Buffers used: citrate buffer (pH 4–6), Tris–HCl buffer (pH 7–8), and sodium carbonate-bicarbonate buffer (pH 9). All buffer concentrations were 0.1 M. Assays were conducted at 25 °C. Values represent the mean ± SD (*n* = 3) from three independent experiments.

To evaluate the rSM519 sensitivity to inhibitors, assays were conducted in the presence of known protease inhibitors (EDTA, PMSF, pepstatin A, and 2-mercaptoethanol). As a control, fibrinolytic activity without the addition of an inhibitor was considered 100% (Figure 7). Although the results with all tested inhibitors showed a statistically significant reduction in fibrinolytic activity compared with the control, this reduction was more severe with EDTA and PMSF, leaving approximately 60% residual activity. Therefore, it is suggested that the enzyme is a serine metalloprotease. This classification aligns with findings reported for the native enzyme.^18^

**Figure 7:**
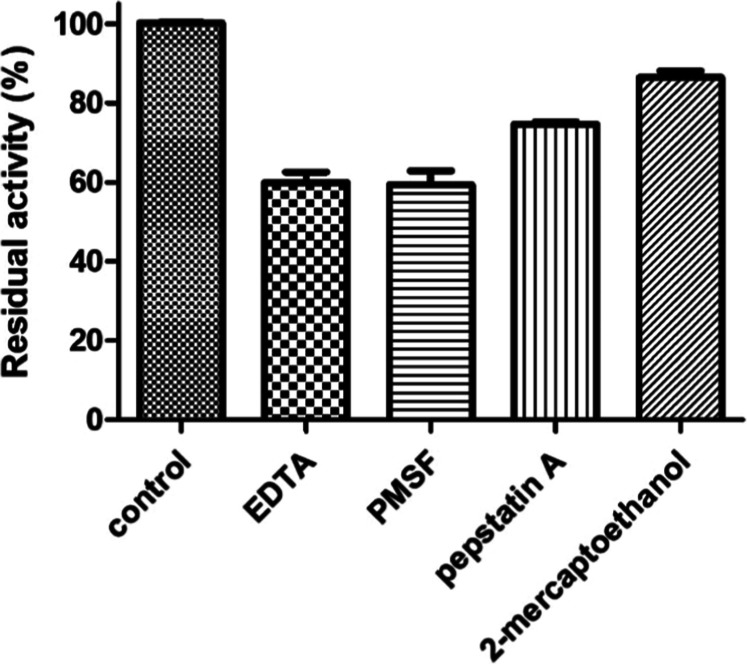
Effect of inhibitors on the fibrinolytic activity of rSM519. The effect of inhibitors on enzyme activity was measured after 1 h of incubation at 37 °C. The reaction was conducted at optimal pH 9.0. The inhibition level was expressed as a percentage of remaining activity compared to the control activity without an inhibitor. Values represent the mean ± SD of experiments (*n* = 3).

Metalloproteases, the most diverse group within proteases, require a divalent metal ion for their activity and are inhibited by chelating agents like EDTA. Most metalloproteases feature the His-Glu-Xaa-Xaa-His (HEXXH) motif, identified by X-ray crystallography as part of the metal-binding site, typically zinc.^43^ Serine proteases, a prominent subgroup, are not affected by chelating agents. On the other hand, PMSF inhibits the activity of certain proteases by sulfonating the crucial serine residue in the active site, leading to inactivation.^44^ Serine metalloproteases exhibit properties of both serine proteases and metalloproteases.^34^

Many microbial fibrinolytic enzymes, including nattokinase and subtilisin DFE, are serine proteases,^10,31,45^ while enzymes from Streptomycessp.,^38^ Neurospora sitophila,^46^ Bacillus circulans,^47^ and S. marcescens subsp. Sakuensis^3^ have been reported as serine metalloproteases, as their activities were inhibited by both protease inhibitors and metal chelators.

To evaluate the effect of metal ions on the fibrinolytic activity of rSM519, the assay was conducted by incubating the enzyme in the presence of different metal ions. According to Table 2, results for rSM519 were similar to those observed for the native enzyme: Mn^2+^ continued to enhance the enzymatic activity, showing a residual activity of 359%, while Cu^2+^ caused a 65% loss in activity. Overall, these analyses demonstrate that recombinant expression in a heterologous system preserved the characteristics of the enzyme, without causing significant changes in its physicochemical parameters.

**Table 2:** Effect of Metal Ions on rSM519 Activity^a^

ion	residual activity (%)	*p*-value
	10 mM	
control	100 ± 2.66	
Na^+^	90.60 ± 0.33	<0.05
Ca^2+^	79.21 ± 1.00	<0.001
Fe^2+^	69.80 ± 0.67	<0.001
Cu^2+^	34.16 ± 3.66	<0.001
Mg^2+^	100.50 ± 4.99	ns
Zn^2+^	63.86 ± 0.00	<0.001
K^+^	90.10 ± 0.33	<0.05
Mn^2+^	359.42 ± 2.00	<0.001

a The data represent mean ± SD (*n* = 3). *p*-values derived from the Student’s *t*-test compared to the control group values.

#### 3.2.4 In Vitro Hemolysis Assay

The hemolysis test conducted after cloning and expression of rSM519 in a heterologous system demonstrated no hemolytic activity on blood agar plates (Figure 8). These combined characteristics lead to the conclusion that rSM519 has potential as a therapeutic agent for the treatment and prevention of thrombosis. Further tests are necessary to assess the enzyme’s cytotoxic effects under different experimental conditions.

**Figure 8:**
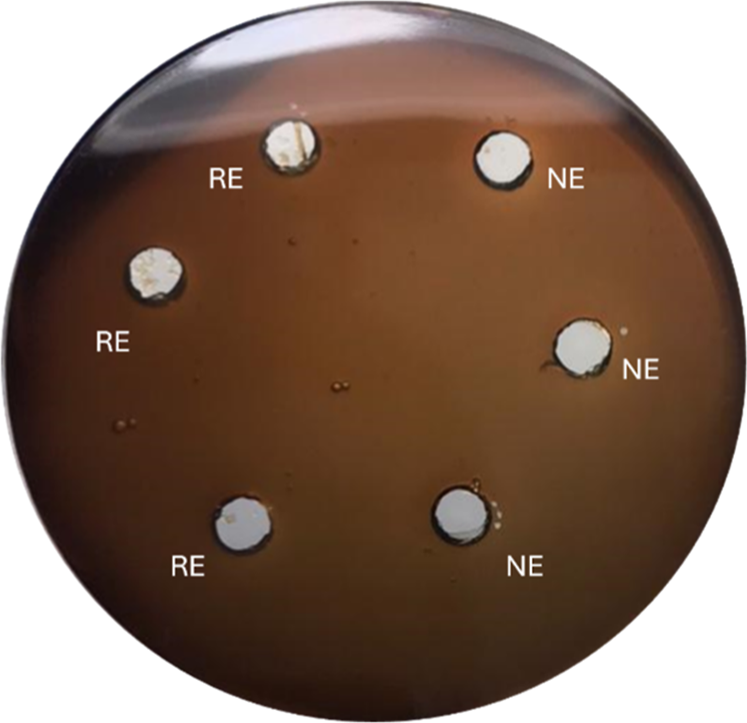
In vitro hemolysis assay of S. marcescens CBAM 519. The assay was performed on blood agar plates and incubated at 37 °C for 3 days. Native enzyme (NE) and recombinant enzyme (RE) were tested. The protein concentration in each well was 0.25 mg/mL (*n* = 3).

The comparative analysis in Table 3 evaluates the biochemical features of S. marcescens CBAM519 fibrinolytic enzyme in its native form^18^ (directly purified from the bacterial strain) versus the recombinant version (heterologously overexpressed in E. coli BL21­(DE3)). In general, recombinant expression, as previously discussed, can yield a protein with distinct characteristics compared to its native form, primarily due to alterations in protein folding. In this particular case, the protein was produced in inclusion bodies and subsequently refolded in vitro while maintaining its enzymatic activity. These observed differences may stem from various factors, including production rates, bacterial cytosol conditions, or other host-specific constraints. Importantly, heterologous expression can be optimized through multiple strategies, such as vector selection, culture media adjustment, host engineering, and fermentation mode modification. For instance, if the protein requires specific post-translational modifications, then an alternative host capable of performing these biochemical reactions should be considered. Optimization of the rSM519 production process, particularly focusing on improving the yield and functionality, represents a key objective for the next phase of this project.

**Table 3:** Comparative Features of Native and Recombinant Fibrinolytic Enzymes from Serratia marcescens CBAM 519

parameter	native enzyme^18^	recombinant enzyme (rSM519)—this study
molecular weight	∼56 kDa	∼56 kDa
optimum pH	9.0	9.0
pH stability	stable from pH 5.0 to 9.0 for 24 h	stable from pH 5.0 to 9.0 for 24 h
optimum temperature	37 °C	37 °C
plasminogen activation	absent	absent
fibrinogenolytic activity	rapid degradation of α, β and γ chains	rapid degradation of α and β chains; γ chain spared
effect of Mn^2+^	strong activation	strong activation
effect of Cu^2+^ and Fe^2+^	inhibitory	inhibitory
protease class	serine-metalloprotease (sensitive to PMSF and EDTA)	serine-metalloprotease (sensitive to PMSF and EDTA)
hemolytic activity	absent	absent
plasmin-like profile	yes, degrades fibrin directly	yes, degrades fibrin directly

## 4 Conclusion

This pioneering study successfully demonstrated the cloning and heterologous expression of fibrinolytic protease rSM519 from S. marcescens in E. coli. The recombinant enzyme retained its biochemical and functional properties, closely mirroring those of the native form, including fibrinolytic and fibrinogenolytic activities and plasmin-like behavior. The enzyme demonstrated efficient fibrin degradation without requiring plasminogen activation—an important advantage over conventional thrombolytic agents such as tPA, urokinase, and streptokinase, which are associated with high costs, limited specificity, and adverse effects. Its recombinant expression in E. coli provides a scalable and cost-effective alternative to traditional production systems. Moreover, the absence of hemolytic activity further supports its potential for biomedical use. Moving forward, future research should focus on in vivo thrombolysis models to confirm the therapeutic efficacy and safety of rSM519 under physiological conditions. Additionally, comprehensive cytotoxicity assays using human cell lines are essential to evaluate its biocompatibility and to anticipate potential side effects. To enable translational and industrial applications, efforts should also be directed toward process optimization and scale-up strategies, including fermentation parameter refinement and cost-effective purification workflows. These steps are essential to advance rSM519 from a promising laboratory enzyme to a clinically and commercially viable biopharmaceutical.
